# Comparison of Arthroscopic Partial Meniscectomy to Physical Therapy following Degenerative Meniscus Tears: A Systematic Review and Meta-analysis

**DOI:** 10.1155/2020/1709415

**Published:** 2020-03-03

**Authors:** Juntan Li, Wannan Zhu, Xiang Gao, Xu Li

**Affiliations:** ^1^Department of Orthopedics, The First Hospital of China Medical University, 155 Nanjing North Street Shenyang 11001, China; ^2^College of Rehabilitation and Sports Medicine, Jinzhou Medical University, No. 40, Sec.3, Songpo Rd. Linghe Dist. Jinzhou, Liaoning 121000, China

## Abstract

**Objective:**

To compare the effectiveness of arthroscopic partial meniscectomy (APM) and physical therapy (PT) for degenerative meniscus tears.

**Method:**

We conducted a literature search through PubMed, Embase, the Cochrane Central Register of Controlled Trials, and ClinicalTrials.gov. Randomized controlled trials in adults with degenerative meniscal tears without symptoms of locking were considered for inclusion. Two researchers independently performed the literature search, assessed the risk of bias, and selected eligible studies. The primary outcome was function at different follow-up time points and the secondary outcome was pain at different follow-up time points.

**Results:**

We included 6 randomized controlled trials, with a total of 1006 participants, among which 495 were in the APM group and 511 were in the PT group. We found a small benefit in functional outcomes in the APM group until the 12 months follow-up time point (SMD = 0.20; 95%CI = 0.0‐0.33; *p* = 0.002; *I*^2^ = 34%), but no significant differences in function between groups at the 24-month follow-up time point (SMD = 0.12; 95%CI = −0.04 − 0.28; *p* = 0.002; *I*^2^ = 34%), but no significant differences in function between groups at the 24-month follow-up time point (SMD = 0.12; 95%CI = −0.04 − 0.28; *p* = 0.002; *I*^2^ = 34%), but no significant differences in function between groups at the 24-month follow-up time point (SMD = 0.12; 95%CI = −0.04 − 0.28; *p* = 0.002; *I*^2^ = 34%), but no significant differences in function between groups at the 24-month follow-up time point (SMD = 0.12; 95%CI = −0.04 − 0.28;

**Conclusion:**

In the treatment of degenerative meniscus tears, APM yielded better functional and pain outcomes compared with physical therapy in the short term until 12 months, but there were comparable results for pain and functional outcomes between the groups at the 24 months follow-up time point.

## 1. Introduction

The meniscus is a fibrocartilage structure inside the knee joint [1] that has important load transmission and load-bearing functions [2]. The meniscus also increases the stability and congruity of knee kinematics because of its unique concave surface, which accommodate the convex femoral condyle [3, 4].

Meniscus tears can be traumatic or degenerative, with traumatic meniscus tears usually occurring in younger patients as a result of being injured during sport. This type of tear usually consists of symptoms of locking and catching due to the unstable meniscus becoming stuck between the medial and lateral condyles. Surgery may be conducted to excise the unstable meniscus, which is known as arthroscopic partial meniscectomy (APM). According to a recent survey, APM has become the most common orthopedic surgery [5], with 465,000 APM performed per year in the United States alone [6], and a global cost of billions of dollars per year [7].

Degenerative meniscus tears (DMT) often occur in the elderly and are accompanied by cartilage damage and osteoarthritis [8]. This type of tear may be symptomatic or asymptomatic and according to a survey by Englund et al. [9], 61% of asymptomatic patients older than 50 years have evidence of a meniscus tear on magnetic resonance imaging. Depending on the symptoms and duration of meniscus tears, surgeons may opt for different treatments, including APM [10] or conservative treatments such as physical therapy (PT) or pharmacological treatment.

However, the decision whether or not to pursue APM for degenerative meniscus tears is still unclear. Physical therapy (PT) has been shown to be a very effective treatment method for patients with varying degrees of osteoarthritis [11, 12]. Degenerative meniscus tears are usually accompanied by mild osteoarthritis, and therefore PT treatment could reduce pain in the knee joint by improving the function of the knee and strengthening the muscles around the knee. The latest guideline also suggest that PT should be a first-line treatment for DMT and that APM should be performed to improve the function and pain of the knee joint if physical therapy is not effective [13, 14]. However, it should also be considered that arthroscopic surgery may cause some rare but serious complications [15].

Therefore, it is still unknown whether APM or PT is more effective for treating patients with a degenerative meniscus tear. The purpose of this meta-analysis was to summarize data from multiple randomized controlled trials and derive evidence-based medical guidelines for the treatment of degenerative meniscus injuries. We hypothesized that APM provides superior functional recovery and pain recovery than PT in patients with degenerative meniscus tears.

## 2. Methods

### 2.1. Search Criteria

This meta-analysis was conducted in accordance with the preferred reporting items for systematic reviews and meta-analysis (PRISMA) guidelines [16, 17] and the protocol for this meta-analysis was registered in PROSPERO (registration number: CRD42019125653). We performed a database search in PubMed, Embase, the Cochrane Central Register of Controlled Trials, and ClinicalTrials.gov from their inception to January 1, 2019, using the following terms: “Arthroscopic Meniscectomy” and “physical therapy.” We also performed a search of the reference lists of included articles and the “cited by” articles to identify any additional relevant articles.

### 2.2. Selection of Studies

The criteria for inclusion in the meta-analysis were as follows: (1) randomized controlled trials, (2) at least one group of participants in the study with degenerative meniscal injury that received either arthroscopic partial menisectomy or physical therapy, (3) patients had to be >40 years old, and (4) study had to contain at least one outcome measure. Exclusion criteria included the following: (1) observational studies, (2) diagnosis of obstructive meniscal tears, (3) meniscal repair or concurrent anterior cruciate ligament lesion, (4) sham surgery, and (5) review or case report articles. Two members of the study group independently assessed whether the retrieved articles met these inclusion and exclusion criteria by screening the full texts. Any disagreements between the two authors were settled by discussion.

### 2.3. Quality Assessment

Each article was independently evaluated by two of the study authors. If there was a dispute, an in-person discussion was held to reach consensus. Methodological quality of included studies was assessed for risk of bias using the following criteria recommended by the Cochrane Bias Methods Group [18]: the randomization procedure; allocation concealment; blinding of patients, surgeons, and outcome assessors; selective outcome reporting; and incomplete outcome data. The two first authors independently made a judgement of high, low, or unclear risk of bias. The Grading of Recommendations Assessment, Development and Evaluation (GRADE) method was used to assess the evidence of each outcome [19].

### 2.4. Data Extraction and Analysis

The primary outcome for this meta-analysis was patients' functional outcome, which was measured using various patient-reported outcome measures (PROMs), such as the Western Ontario and McMaster Universities Osteoarthritis Index (WOMAC), the Knee injury and Osteoarthritis Outcome Score (KOOS), the Lysholm Knee Scoring Scale (LKSS), and the International Knee Documentation Committee (IKDC) questionnaire. The secondary outcomes were pain, as measured using several pain measurement scales, such as the visual analog scale (VAS), the VAS for weight bearing, and the KOOS pain subscale score. Adverse events such as cardiovascular, neurological, internal medical conditions, venous thromboembolism, reactive arthritis, and surgical site infection were counted in each group.

Two members of the study team independently extracted the data from each study included in this review and all data was stored in Excel. The extracted data included numbers of participants in each group, sex, age, body mass index (BMI), time to follow up, study outcome measurements, osteoarthritis progression, crossover rate, and adverse events. The original authors of the articles were contacted if the article did not contain the necessary data for meta-analysis. Each study evaluated a variety of postoperative functional scales, so if an article used two functional outcome measures we extracted the primary outcome measure only. The same method was followed for data extraction of pain outcome measures. Extracted outcome data were stratified by follow-up time (3 months, 6 months, 12 months, and 24 months). The mean and standard deviation (SD) of follow-up scores were extracted from both the APT group and PT group. When the SD was not available, it was calculated using the provided 95% confidential interval (95% CI) or estimated using the interquartile range [20]. When follow-up scores were not available, the mean and standard deviation (SD) of the change from baseline to follow-up were extracted from the studies for the APT and PT groups.

Standard mean differences (SMD) with 95% CIs were calculated to pool the results for continuous outcomes that were measured using different scales. Risk ratios (RR) with 95% CIs were calculated for dichotomous measures. The significance level for all analyses was set at *p* < 0.05. Heterogeneity was assessed using the *I*^2^ value. Significant heterogeneity was established for *I*^2^ values > 50% and *p* < 0.05. A fixed effects model was utilized for outcome data without significant heterogeneity and a random effects model was used for data with significant heterogeneity. All data analysis was conducted using Review Manager Version 5.3.

## 3. Result

**Figure 1:**
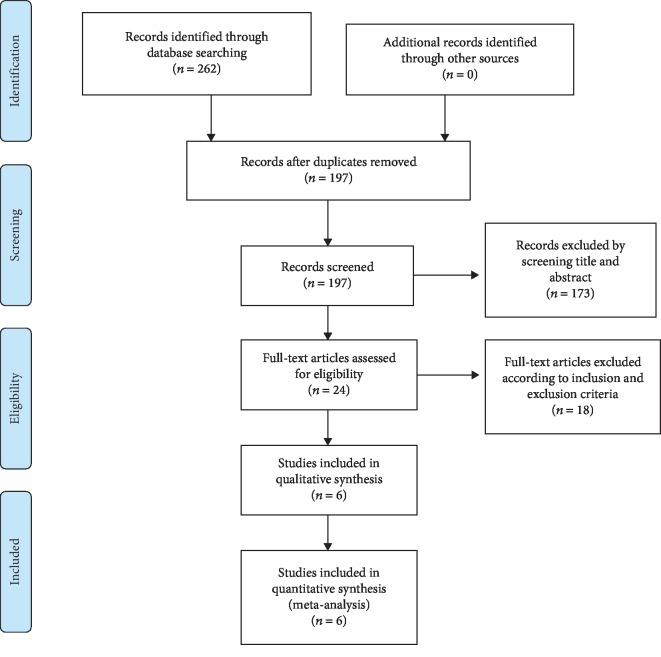
PRISMA flow chart.

A flow chart of the included and excluded articles is presented in Figure 1. Our database search yielded a total of 262 articles, of which 197 remained after excluding duplicate texts. Screening the titles and abstracts according to the inclusion and exclusion criteria resulted in 24 remaining articles, of which 18 further articles were excluded after reading the full text, resulting in a total of 6 randomized controlled trials [21–26] being included in this review and meta-analysis.

### 3.1. Characteristics of the Included Studies

The characteristics of the included studies are presented in Table 1. The six included studies had a total of 1006 participants, with 495 in the APM group and 511 in the PT group. The mean age in the APM group was 56.4 years old, compared to 55.9 in the PT group. Approximately half of the participants were female in both groups (52.5% in APM group, 51.9% in PT group). All of the included studies were RCTs, with level 1 evidence. Meniscus injuries in the included studies were degenerative, with no symptoms of locking or catching. All of the included studies specified their PT protocols in the article. In the study of Herrlin et al. [21], all participants in the APM group received postoperative PT, while in the other 5 studies postoperative PT was conducted in the APM group only if deemed necessary.

### 3.2. Risk of Bias

**Figure 2:**
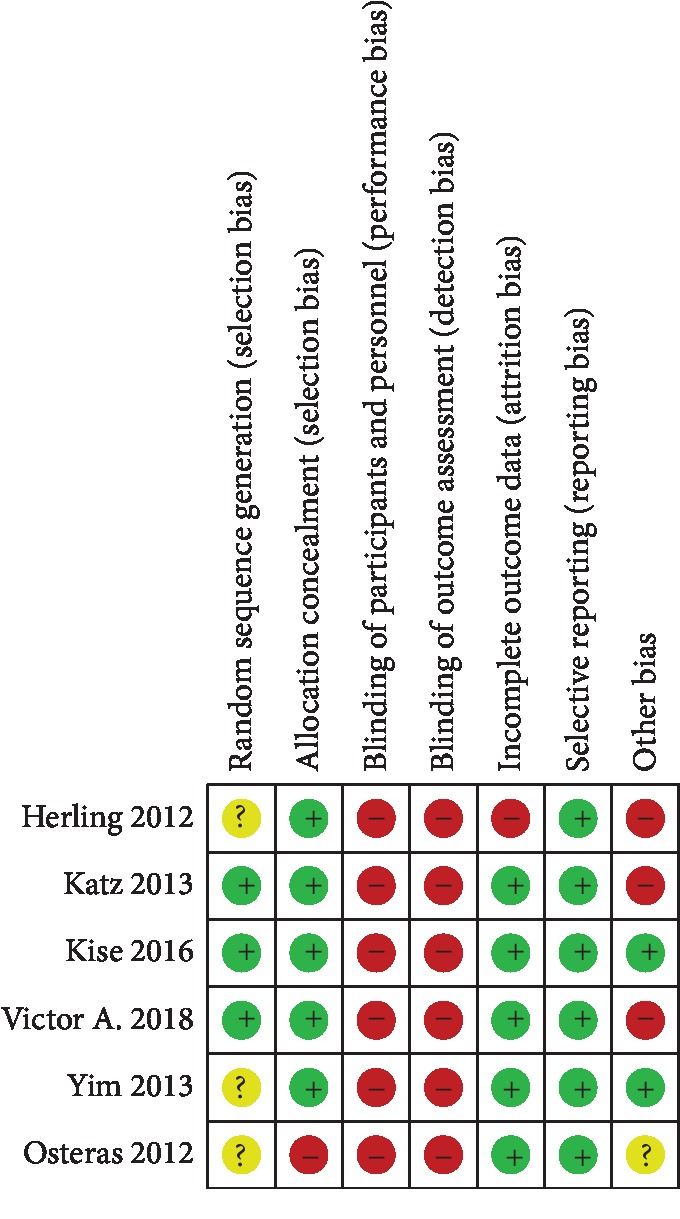
Risk of bias assessment.

Risk of bias assessments of the included studies are presented in Figure 2. Since double blinding was not possible due to one group receiving surgery and the other group performing physical therapy, all of the included studies were considered as high risk for methodological bias. Three studies used appropriate randomization procedures, but the other three studies did not mention their randomization procedure. All of the included studies fail to blind their outcome assessments. The rate of loss to follow-up was high in one of the included articles, which may have caused bias due to attrition.

### 3.3. Knee Function

**Figure 3:**
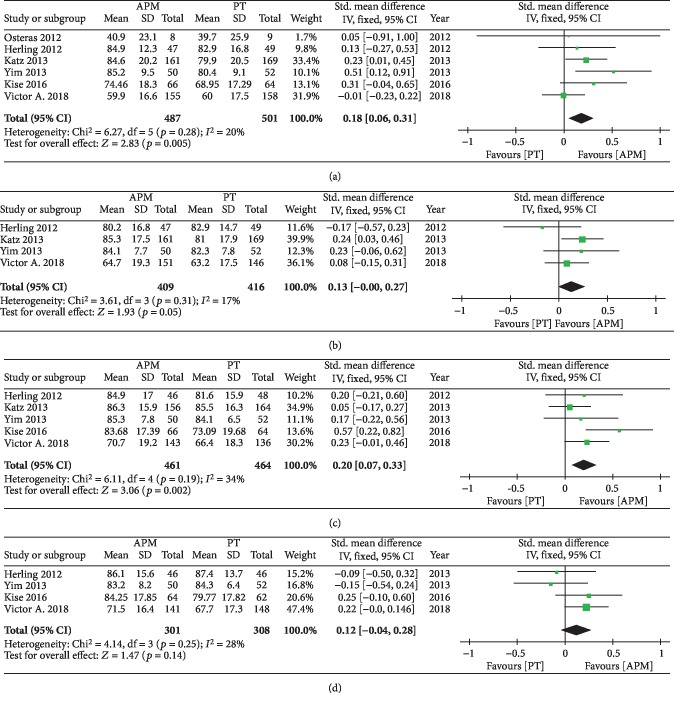
Forest plot of functional outcomes at different follow-ups.

Different outcome measures were used across the six included studies, with the KOOS used to evaluate knee function in three studies [21, 24, 27], the LKSS used in one study [26], and the IKDC used in one study [25]. Therefore, we calculated the SMD for the primary outcome of knee function across different PROMs, with a forest plot of the pooled results presented in Figure 3.The pooled results demonstrated differences between the two groups at 3 months (SMD = 0.18; 95%CI = 0.06 − 0.31; *p* = 0.005; *I*^2^ = 20%), 6 months (SMD = 0.13; 95%CI = 0.00 − 0.27; *p* = 0.05; *I*^2^ = 17%), and 12 months (SMD = 0.20; 95%CI = 0.0 − 0.33; *p* = 0.002; *I*^2^ = 34%) of follow-up, with participants who received APM achieving better functional improvements compared with the PT group. However, there were no significant differences between groups at 24 months (SMD = 0.12; 95%CI = −0.04 − 0.28; *p* = 0.14; *I*^2^ = 28%).

### 3.4. Knee Pain

**Figure 4:**
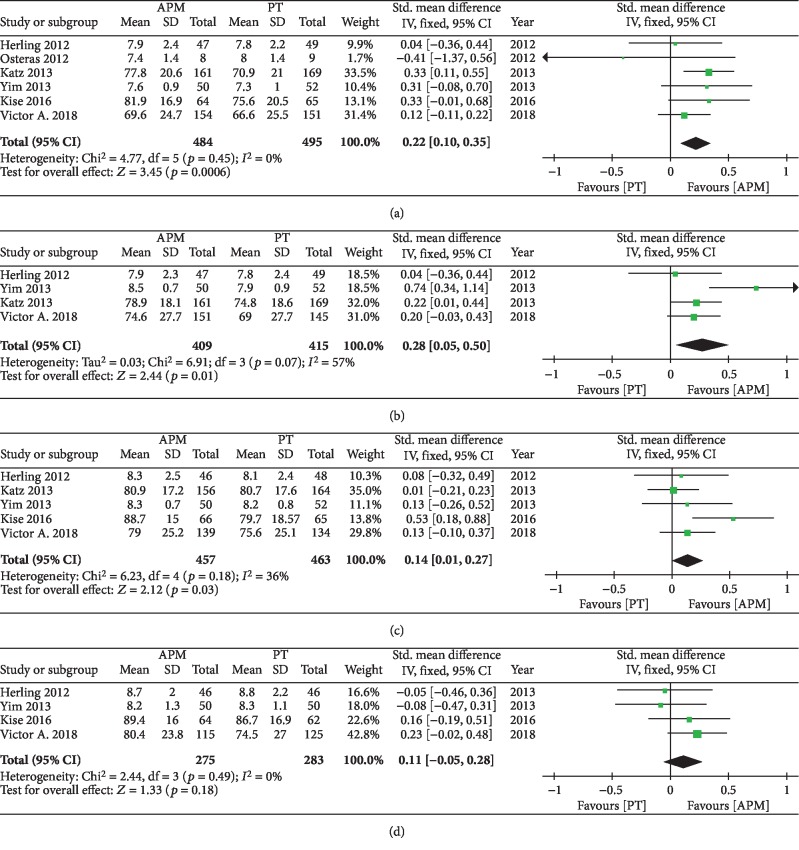
Forest plots of pain outcomes at different follow-ups.

Four studies used the VAS scale to evaluate knee pain [21, 24–26] and two studies used the KOOS pain subscale [23, 27]. A forest plot of the pooled results is presented in Figure 4. The pooled results revealed significantly improved pain recovery in the APM group at 3 months (SMD = 0.22; 95%CI = 0.10 − 0.35; *p* < 0.0006; *I*^2^ = 0%), 6 months (SMD = 0.28; 95%CI = 0.05 − 0.50; *p* = 0.01; *I*^2^ = 57%), and 12 months (SMD = 0.14; 95%CI = 0.01 − 027; *p* = 0.03; *I*^2^ = 36%) of follow-up. However, there were no significant differences at the 24-month follow-up time point (SMD = 0.11; 95%CI = −0.05 − 0.28; *p* = 0.18; *I*^2^ = 0%).

### 3.5. Other Outcomes

Two studies evaluated general health at 24 months of follow-up, one using the SF-36 score and one using the RAND-36 score [23, 25]. When we performed a meta-analysis using the data from these two articles, the general health of the APM group was significantly better than the PT group (SMD = 0.33; 95% CI = 0.14 − 0.53; *p* = 0.0008; *I*^2^ = 0%) at 24 months.

Three studies reported the occurrence of adverse events until the final follow-up time point [23, 25, 27]. Kise et al. did not report any serious adverse events in either group [23], while Katz et al. reported 3 serious and 15 nonserious adverse events in the APM group and 2 serious and 13 nonserious adverse events in the PT group [27]. Van de Graaf et al. reported 9 serious and 9 nonserious adverse events in the APM group, compared with 8 serious and 4 nonserious adverse events in the PT group [25].

Five studies reported the crossover rate of participants who were randomized to the PT group but elected to undergo APM surgery during the follow-up observation period. The overall average crossover rate across these five studies was 26.0%.

## 4. Discussion

This article is aimed at comparing the outcomes of arthroscopic partial meniscectomy (APM) and physical therapy (PT) for patients with degenerative meniscal tears. The results of our meta-analysis demonstrate that APM leads to more effective recovery in functional and pain outcomes in the short term when compared with PT. We found that APM was superior to PT in both functional and pain-related PROMs at 3 months, 6 months, and 12 months of follow-up. However, when the follow-up time was extended to 24 months, we did not find a significant difference between the two groups. This indicates that APM should be considered a treatment for degenerative meniscal tears that can provide better results than PT in the short term. However, these results need to be interpreted carefully due to the small sample of total participants and small number of included studies.

Meniscectomy is a globally accepted procedure among orthopedic surgeons and APM has become the most common orthopedic surgery, with nearly 2 million procedures performed each year [7]. According to a study by Roos et al., radiographic osteoarthritis was 14 times more common in people two decades after having a total meniscectomy compared to age-matched and gender-matched controls [28]. Therefore, the long-term effectiveness of APM is still questionable, especially following the publication of several recent clinical studies that claim that meniscectomy should not be recommended for all people and that it may cause serious problems [29, 30]. A number of previous studies have shown that arthroscopic knee surgery may cause aggravation of osteoarthritis [31, 32], but neither of the two studies included in this meta-analysis that examined this outcome found evidence of OA exacerbation [21, 25].

There have been a number of articles comparing APM with conservative treatment that did not pay close attention to the various follow-up time points and had a relatively short follow-up time period [33, 34]. A study by Van de Graaf et al. [35] concluded that there were differences after treatment between the two groups at 3 months and 6 months, but not at 12 months. In this paper, a new search was conducted to identify the latest literature, which allowed for the inclusion of a new multicenter RCT study. We found that there was a small improvement in knee function and pain among APM patients compared to PT patients at 3, 6, and 12 months, but there were no differences at 24 months. However, the reasons for this change over time are unknown. We believe that the population of the included studies may have been responsible for this change, as degenerative meniscal tears are usually concomitant with osteoarthritis and cartilage injury. Therefore, the positive effects of APM were gradually attenuated over time due to progressing osteoarthritis, which resulted in no significant difference between the two groups at the long-term follow-up time point.

The results of this study reveal the potential short-term advantages provided by APM. Although physical therapy can improve knee function and reduce pain, five studies of the six included studies reported that some participants were not satisfied with the effects of physical therapy and crossed over from the PT group to the APM group during the study, with the highest reported rate of crossover reaching nearly 30%. As a result of the anatomical characteristics of the meniscus, only one-third of the meniscus receives an adequate blood supply in adults [36]. While physical therapy may result in complete healing of a stable tear, it can also lead to a reparable or irreparable tear if the tear has progressed due to a traumatic history, especially in populations participating in demanding activities. Our results suggest that APM is a logical treatment for that population, unlike previous articles that regarded APM as nonbeneficial or even leading to serious consequences. However, further research is needed to find out who is not likely to respond to physical therapy to determine reasonable indications for APM surgery.

### 4.1. Limitations

There are several limitations to note in this study. First, this paper only included six studies due to the current number of randomized controlled trials in this research area. Although these six studies are all level 1 studies in terms of level of evidence, the small number of studies and overall sample size limits our ability to draw broad conclusions. Different studies included in this meta-analysis adopted different PROM scales. When using GRADE to evaluate evidence, the use of different methods of measuring outcomes limits the ability of the included studies to be classified as high-level evidence. Due to the lack of blinding and potential selection bias, the overall level of evidence as measured according to the GRADE method is low to very low.

Second, participants crossed over from the PT group to the APM group in almost every study included in this analysis. The data of these participants were not retained and further information on additional surgeries was unavailable.

Third, the inclusion criteria of the article did not classify osteoarthritis. The symptoms of mild osteoarthritis are much different than those of severe osteoarthritis, which may influence treatment and recovery. The studies in this meta-analysis included all levels of osteoarthritis and we did not conduct a subgroup analysis. Finally, although there is significant evidence that shows that PT can restore function of the knee, the included studies all utilized different PT regimens, and one article did not describe its PT regimen.

## 5. Conclusion

We found a small but statistically significant effect favoring APM over PT for physical function and pain outcomes up to the 12-month follow-up time point. However, APM and physical therapy yielded comparable results at the 24-month follow-up time point.

## Figures and Tables

**Table 1:** Characteristic of included studies.

Study	Publication year	Follow-up (months)	OA grade	Outcome measurement	Cross-rate (%)	Group	Numbers	Age	Female percent	Adverse event
Osteras et al.	2012	3	K-L 0-2	KOOS VAS pain	NR	APM	8	52.7 ± 7.2	37.5%	NR
						PT	9	47 ± 10.4	11.1%	NR

Herrlin et al.	2012	6, 24, 60	Ahlback 0-1	KOOS Lysholm VAS pain Tegner	28.2	APM	47	56.4	40.4%	NR
						PT	49	55.9	38.8%	NR

Katz et al.	2013	6, 12	K-L 0-3	WOMAC SF-36 KOOS pain	30.2	APM	161	59.0 ± 7.9	55.9%	18
						PT	169	57.8 ± 6.8	57.4%	15

Yim et al.	2013	3, 6, 12, 24	K-L 0-1	Lysholm VAS pain	1.9	APM	50	57.6 ± 11.0	82%	NR
						PT	52	54.9 ± 10.3	76.9%	NR

Kise et al.	2016	3, 12, 24	K-L 0-3	KOOS-4 SF-36	18.6	APM	70	48.9 ± 6.1	38.6%	0
						PT	70	50.2 ± 6.2	38.6%	0

Van de graaf et al.	2016	3, 6, 12, 24	K-L 0-3	IKDC VAS pain SF-36 EQ-5D Tegner	29	APM	159	57.6 ± 6.5	50.3%	18
						PT	162	57.3 ± 6.8	50%	12
